# Exploring causal networks of bovine milk fatty acids in a multivariate mixed model context

**DOI:** 10.1186/1297-9686-46-2

**Published:** 2014-01-17

**Authors:** Aniek C Bouwman, Bruno D Valente, Luc L G Janss, Henk Bovenhuis, Guilherme J M Rosa

**Affiliations:** 1 Animal Breeding and Genomics Centre, Wageningen University, P.O. Box 338, 6700 AH Wageningen, The Netherlands; 2 Department of Animal Sciences, University of Wisconsin, Madison, WI 53706, USA; 3 Faculty of Science and Technology, Department of Molecular Biology and Genetics, University of Aarhus, DK-8830 Tjele, Denmark; 4 Department of Biostatistics and Medical Informatics, University of Wisconsin, Madison, WI 53706, USA

## Abstract

**Background:**

Knowledge regarding causal relationships among traits is important to understand complex biological systems. Structural equation models (SEM) can be used to quantify the causal relations between traits, which allow prediction of outcomes to interventions applied to such a network. Such models are fitted conditionally on a causal structure among traits, represented by a directed acyclic graph and an Inductive Causation (IC) algorithm can be used to search for causal structures. The aim of this study was to explore the space of causal structures involving bovine milk fatty acids and to select a network supported by data as the structure of a SEM.

**Results:**

The IC algorithm adapted to mixed models settings was applied to study 14 correlated bovine milk fatty acids, resulting in an undirected network. The undirected pathway from C4:0 to C12:0 resembled the *de novo* synthesis pathway of short and medium chain saturated fatty acids. By using prior knowledge, directions were assigned to that part of the network and the resulting structure was used to fit a SEM that led to structural coefficients ranging from 0.85 to 1.05. The deviance information criterion indicated that the SEM was more plausible than the multi-trait model.

**Conclusions:**

The IC algorithm output pointed towards causal relations between the studied traits. This changed the focus from marginal associations between traits to direct relationships, thus towards relationships that may result in changes when external interventions are applied. The causal structure can give more insight into underlying mechanisms and the SEM can predict conditional changes due to such interventions.

## Background

In animal breeding and genetics, relationships between traits are traditionally studied using multi-trait mixed models [1]. Such models do not allow for recursive relationships between traits that are generally present in biological systems. Structural equation modelling (SEM) is a statistical technique for testing and estimating such recursive relationships [2-4]. Gianola and Sorensen [5] described SEM in a quantitative genetics context in order to account for possible feedback or recursive relations among traits in multi-trait mixed models settings. In most applications of SEM in animal breeding and genetics, only few hypothesized networks are typically tested and compared, and those that best fit the data are declared as most plausible [6-10]. Although such an approach avoids the computational challenges involved in testing every possible network, it does not explore the full space of possible networks. However, data driven exploration of the space is possible using the Inductive Causation (IC) algorithm [11].

The IC algorithm is based on conditional independencies tests, such that under multivariate normality, it can be implemented by using partial correlations tests. When all partial correlations between a pair of traits are non-null for each conditioning subset of traits (i.e., they are dependent conditionally on all possible sets of other traits), then a direct causal relation between this pair of traits is declared. When a partial correlation between two traits is null (i.e., they are independent conditionally on at least one set of other traits), then there is no direct causal relation between this pair of traits. Therefore, partial correlations can be explored to study how a set of traits is causally related and this can be qualitatively represented by a graph or network [3]. If the resulting network is completely directed, it can be used as a causal structure of a SEM, and the magnitude of causal relationships among traits (represented by structural coefficients) can be estimated by fitting such a model. Furthermore, visualization of the causal relationships among variables on a graph could help understand and interpret complex biological systems, while their quantification allows prediction of outcomes of external interventions applied to such a causal network.

The inferred structural coefficients associated with connections between traits in a network only carry a causal interpretation under specific causal assumptions. For example, structural coefficients inferred from a SEM with an acyclic causal structure and independent residuals only keep their causal meaning under the assumption that there are no hidden causal effects that have a direct influence on two or more traits in the network. In livestock, removing such confounding effects can be achieved by performing randomised experiments. However, most livestock data come from non-randomised field studies and are prone to the influence of several sources of systematic variation. When measured, the confounding generated by these systematic sources of variation can be controlled by correcting for them in a model. One example of hidden factors that may affect two or more traits in the network is correlated genetic effects. Thus, the genetic covariances are background sources of phenotypic covariances among traits that confound not only the inference of causal effects between pairs of traits, but also the search for causal structures, because algorithms may interpret such covariances as due to causal relations among phenotypes. Therefore, Valente et al. [12] proposed to use the inferred residual (co)variance matrix of a standard multi-trait mixed model (which represents the covariance matrix among traits conditionally on the genetic confounders) as input for the IC algorithm, instead of the observed data, when searching for causal structures in mixed effects settings. Valente et al. [12,13] used simulated data to show that applying the IC algorithm to the posterior distribution of the residual (co)variance matrix of a multi-trait mixed model recovered the correct network, and Valente et al. [13] used the methodology on real data from quails to study causal networks involving five traits.

Here, we applied the same approach to a set of 14 highly correlated milk fatty acids to analyse their causal relations. Fatty acids are important components in human diets with either beneficial or unfavourable effects on human health, depending on the fatty acid. Studying causal relations between bovine fatty acids in milk can provide valuable information about the synthesis of fatty acids, which could be useful for approaches aimed at changing the fatty acid composition of dairy products and ultimately at improving human health. Since a considerable amount of knowledge about the synthesis of fatty acids is available, the network obtained from the adapted IC algorithm can be compared to known biological pathways. However, the network may also reveal new relations that could confirm existing hypotheses or create new ones. The known biological pathways include *de novo* synthesis, biohydrogenation and desaturation of milk fatty acids. Most of these pathways are reflected in the results of analyses that involve phenotypic and genetic correlations between milk fatty acids [14-16], clustering techniques [17,18], or principal component analysis [19]. These studies suggest that certain fatty acids have a common origin, but they cannot distinguish between direct and indirect relationships.

Our aim was to explore causal networks between milk fatty acids by applying for the first time the adapted IC algorithm as presented by Valente et al. [12] to 14 highly correlated traits. In addition, the selected network was used as the causal structure of a SEM to quantify the relationships between the milk fatty acids.

## Methods

### Data

Data on the fat composition of winter milk samples from 1902 first-lactation Dutch Holstein Friesian cows were used. The cows were housed on 397 commercial farms throughout the Netherlands. At least three cows between 63 and 282 days in milk were sampled per farm. The pedigree of the cows was supplied by CRV (Cooperative cattle improvement organization, Arnhem, the Netherlands) and included information from the last four generations (4676 animals).

Milk fat composition was measured by gas chromatography (details about the phenotyping are in Stoop et al. [16]). Fourteen fatty acids with the highest concentration in milk fat were considered: even-chain saturated fatty acids C4:0, C6:0, C8:0, C10:0, C12:0, C14:0, C16:0, C18:0, even-chain (*cis9*) monounsaturated fatty acids C10:1, C12:1, C14:1, C16:1, C18:1, and the polyunsaturated fatty acid CLA (conjugated linoleic acid, C18:2*cis9*,*trans11*). Gas chromatography was performed on fat samples and provided relative amounts of fatty acids expressed on a fat basis in g/100 g fat. However, these relative amounts do not properly represent the biological relationships among fatty acids; therefore the fatty acids were expressed on a milk basis in g/kg milk. Table 1 presents the mean and adjusted phenotypic standard deviation for the fatty acids included in this study.

**Table 1:** **Mean and phenotypic standard deviation**^**1 **^**for bovine milk fatty acids (in g/kg milk)**

**Trait**	**Mean**	**σ**_**p**_^**1**^
C4:0	1.53	0.26
C6:0	0.97	0.17
C8:0	0.60	0.11
C10:0	1.32	0.28
C12:0	1.79	0.37
C14:0	5.05	0.77
C16:0	14.27	2.84
C18:0	3.80	0.84
C10:1	0.16	0.04
C12:1	0.05	0.01
C14:1	0.59	0.13
C16:1	0.63	0.19
C18:1	7.87	1.20
CLA	0.17	0.04

^**1**^$\sigma_{p}=\sqrt{\sigma_{a}^{2}+\sigma_{e}^{2}}$.

### Multi-trait analysis

Genetic and residual (co)variances among traits were estimated by fitting a Bayesian multi-trait mixed model that uses latent variables to fit (co)variance structures and a random walk Metropolis-Hastings algorithm to obtain Markov chain Monte Carlo (MCMC) samples for variance components, similar to the latent variable models to estimate genomic (co)variances in Sørensen et al. [20]. Latent variables were used to fit (co)variance structures because most of the milk fatty acids were strongly correlated, both genetically and residually. Fitting a standard multi-trait model for 14 milk fatty acids resulted in convergence issues, but using latent variables to reduce the dimensionality of the data improved convergence of the Bayesian multi-trait mixed model.

Phenotypes were standardised to traits with a mean of 0 and a standard deviation of 1 to reduce scale differences between the milk fatty acids in the multi-trait mixed model. The following multi-trait model was fitted:

y=Xβ+Zu+e,

with the joint distribution of vectors **u** and **e** as:

$$\left[\begin{array}{c} u\\ e \end{array}\right]\sim N\left\{\left[\begin{array}{c} 0\\ 0 \end{array}\right],\left[\begin{array}{c} G_{0}\;⊗\;A\\ 0 \end{array}\;\begin{array}{c} 0\\ R\;⊗\;I \end{array}\right]\right\},$$

where **y** is a vector of phenotypes; **β** is a vector for systematic effects, for each trait the same systematic effects were included: a covariate for days in milk modelled with a Wilmink curve [21], a covariate for age at first calving, a covariate for age at first calving squared, a fixed effect for calving season (June-Aug 2004, Sept-Nov 2004, or Dec 2004-Jan 2005), a fixed effect for sire code (accounting for differences in the genetic level between proven sire daughters and test-sire daughters), and a fixed effect for herd; **X** is a known incidence matrix of **β** on **y**; **u** is a vector of random additive genetic effects; **Z** is a known incidence matrix of **u** on **y**; and **e** is a vector of random residuals. **G**_0_ is the additive genetic (co)variance matrix; **A** is the additive genetic relationship matrix; **R**_0_ is the residual (co)variance matrix; **I** is an identity matrix. The (co)variances between genetic effects and between residuals were modelled employing *k* latent vectors **v**_k_ to model residual (co)variances, and *k* latent vectors **w**_k_ to model genetic (co)variances, such that $e_{i}\sim N\left(\sum_{k}r_{k,i}v_{k},\tau_{e_{i}}^{2}I\right)$ and $u_{i}\sim N\left(\sum_{k}s_{k,i}w_{k},\tau_{u_{i}}^{2}A\right)$, with **v**_*k*_ ~ *N*(0, **I**) and **w**_*k*_ ~ *N*(0, **A**) as standard Normal latent vectors, *r*_*k*,*i*_ and *s*_*k*,*i*_ as regressions or “loadings” on the latent vectors with uniform priors [–∞, ∞], and $\tau_{e_{i}}^{2}$ and $\tau_{u_{i}}^{2}$ as the independent remaining variances for residuals and genetic effects per trait *i*. From the latent variable model, the residual variance for trait *i* is $\sum_{k}r_{k,i}^{2}+\tau_{e_{i}}^{2}$, and the residual covariance between traits *i* and *j* is $\sum_{k}r_{k,i}r_{k,j}$. In a similar manner, the variances $\left(\sum_{k}s_{k,i}^{2}+\tau_{u_{i}}^{2}\right)$ and covariances $\left(\sum_{k}s_{k,i}s_{k,j}\right)$ were obtained for the additive genetic effects.

In order to maintain mixing in the MCMC sampling algorithm, the remaining independent variances $\tau_{e_{i}}^{2}$ and $\tau_{u_{i}}^{2}$ must remain well above 0. Initially, this large set of highly correlated traits resulted in residual and polygenic variances $\tau_{e_{i}}^{2}$ and $\tau_{u_{i}}^{2}$ that were close to 0, thus it was necessary to set a minimum value for them and uniform priors [0.02, ∞] were used on these parameters to achieve this. Because standardised traits were used, these bounds imply that at least 2% of the residual variance for each trait was not explained by residual covariances with other traits, and likewise at least 2% of the genetic variance for each trait was not explained by genetic covariances with other traits. All fixed and random effects (including latent variables) and the regression loadings were conditionally normal, and conditional distributions for variance parameters were scaled inverse Chi square in the MCMC implementation.

The dimension of latent variables *k* is to be pre-set but good indications for this dimension can be obtained by a principal component analysis on the traits analysed, which gives information on the number of latent variables suitable to model the joint (co)variance structure. In order to limit the constraints on the covariance structure, the number of principal components was chosen such that together they explained 90% of the variance. Principal component analysis of the 14 fatty acids showed that the first four principal components explained ~90% of the variance; therefore four latent factors were chosen.

The MCMC software Bayz 2.1 [22] was used for parameter inference. Eight chains of 1 million iterations each were run, with a burn-in of 100 000 for each chain, and a thinning of 1000 iterations. Convergence was checked by visual inspection of the sample trace plots, of posterior density plots and by determining effective sample size using the Coda package in R [23].

### Inductive causation (IC) algorithm

By fitting the multi-trait mixed model described above, the data can be corrected for systematic effects and for genetic (co)variances and thus, inferences regarding the joint distribution of the traits conditionally on genetic and systematic effects can be made. This is important to search for the causal structure using the IC algorithm, because correlated genetic effects are confounding factors, since they are sources of phenotypic correlation due to the genetic background but not due to recursive relations among traits [12]. The relevant information to be used in a causal structure search is in the residual (co)variance matrix that results from a multi-trait mixed model. Therefore Valente et al. [12] proposed to use this matrix as input for the IC algorithm to search for causal networks, instead of using the observed data.

The IC algorithm performs a series of statistical decisions based on partial correlations between traits. The posterior distributions of partial correlations were obtained using the posterior samples of residual (co)variance matrices from the multi-trait analysis and these were then used to test for non-null partial correlations. A partial correlation was declared non-null whenever the highest posterior density (HPD) interval did not include zero. The expected output for the IC algorithm is a partially oriented graph that represents a set of statistically equivalent causal structures.

The IC algorithm consisted of three steps [3]:

#### Step 1

Partial correlations were used to search for edges that connect adjacent variables (two vertices that are endpoints of an edge) to obtain an undirected graph (e.g., *Y*_1_-*Y*_2_). If all partial correlations of two traits conditional on each possible set of other traits were different from zero, an edge was placed between the traits.

#### Step 2

Partial correlations were also used to search for unshielded colliders (three connected variables in a path directed as *Y*_1_ → *Y*_2_ ← *Y*_3_) to orient some edges of the undirected graph provided by step 1. If partial correlations of two non-adjacent traits (e.g., Y_1_ and Y_3_) that have a common adjacent trait (Y_2_) in such an undirected graph are dependent conditional on any possible set that includes the adjacent trait (Y_2_), the edges should be oriented towards the common adjacent trait (Y_2_), such as in *Y*_1_ → *Y*_2_ ← *Y*_3_.

#### Step 3

When possible, remaining undirected edges were oriented in a way that introduced no new unshielded colliders or cycles. This step could only be performed when the graph obtained in step 2 contained unshielded colliders and the orientation followed unambiguously from the graph.

### Structural equation model

Relationships represented by the causal network obtained from the IC algorithm were quantified using a SEM, as in Gianola and Sorensen [5]. The SEM was fitted using Bayesian methods that fit a multi-trait mixed model in software Bayz 2.1 [22], where causal parents (e.g., *Y*_1_ is causal parent of *Y*_2_ in *Y*_1_ → *Y*_2_) of a given trait were considered as covariates in the equations assigned to this trait, and a diagonal residual (co)variance matrix was imposed. Therefore, the following model was fitted:

$$y=\left(\Lambda \;⊗\;I\right)y+X\beta^{*}+Zu^{*}+e^{*},$$

with the joint distribution of vectors **u** and **e** as:

$$\left[\begin{array}{c} u^{*}\\ e^{*} \end{array}\right]\sim N\left\{\left[\begin{array}{c} 0\\ 0 \end{array}\right],\left[\begin{array}{c} G_{0}^{*}\;⊗\;A\\ 0 \end{array}\;\begin{array}{c} 0\\ \Psi_{0}\;⊗\;I \end{array}\right]\right\},$$

where the model was similar to the multi-trait model as described above but with the addition of (**Λ** ⊗ **I**)**y**, where **Λ** is a *t* × *t* (with *t* equal to the number of traits) matrix with 0’s on the diagonal and with structural coefficients or 0’s on the off-diagonals. The causal structure defines which of the off-diagonal entries of **Λ** must be estimated and which ones are set to 0. $G_{0}^{*}$ is the SEM additive genetic (co)variance matrix and **Ψ**_0_ is a diagonal matrix with the SEM residual variances. The residual covariances between the traits in the SEM were assumed to be 0, which confers identifiability to the structural coefficients in the likelihood function. The priors used for the SEM were the same as those used for the multi-trait model.

The SEM was compared with the multi-trait model using the deviance information criterion (DIC) [24]. The DIC takes the trade-off between model goodness-of-fit and corresponding complexity of model into account. Models with smaller DIC are better supported by the data.

## Results

### Multi-trait analysis

Eight independent MCMC chains of the Bayesian multi-trait animal model for the 14 bovine milk fatty acids converged to similar estimates of the variance components, which was confirmed by trace and density plots. The effective sample size for heritabilities, correlations and (co)variance components ranged from 391 to 2431 samples. Posterior means of the heritabilities, genetic correlations and residual correlations between milk fatty acids are shown in Table 2. Fatty acids that are consecutively synthesized *de novo* (e.g., C4:0 and C6:0, C6:0 and C8:0, etc.) generally showed strong positive correlations, both genetically and residually. Residual correlations between medium chain unsaturated fatty acids (C10:1, C12:1, C14:1) and long chain fatty acids (C18:0, C18:1, CLA), and between CLA and C8:0, C10:0, and C12:0 were weak and showed large standard deviations. There were no strong negative correlations between fatty acids.

**Table 2:** **Multi-trait genetic parameters**^**1 **^**for bovine milk fatty acids**^**2,3**^

	**C4:0**	**C6:0**	**C8:0**	**C10:0**	**C12:0**	**C14:0**	**C16:0**	**C18:0**	**C10:1**	**C12:1**	**C14:1**	**C16:1**	**C18:1**	**CLA**
C4:0	**0.53**	0.91	0.83	0.77	0.71	0.87	0.89	0.66	0.50	0.39	0.48	0.50	0.54	0.28
C6:0	0.91	**0.49**	0.94	0.90	0.86	0.93	0.87	0.63	0.63	0.54	0.54	0.49	0.43	0.16
C8:0	0.78	0.91	**0.48**	0.95	0.92	0.93	0.81	0.59	0.67	0.61	0.53	0.45	0.35	0.08
C10:0	0.56	0.76	0.89	**0.43**	0.94	0.92	0.76	0.58	0.64	0.62	0.50	0.40	0.30	0.02
C12:0	0.47	0.66	0.81	0.88	**0.42**	0.89	0.71	0.54	0.62	0.63	0.48	0.37	0.26	-0.01
C14:0	0.56	0.74	0.87	0.91	0.90	**0.39**	0.88	0.65	0.60	0.57	0.56	0.53	0.46	0.16
C16:0	0.87	0.82	0.69	0.49	0.47	0.53	**0.33**	0.55	0.61	0.55	0.69	0.73	0.53	0.31
C18:0	0.80	0.75	0.63	0.44	0.30	0.41	0.69	**0.37**	-0.01	-0.06	0.02	0.13	0.70	0.23
C10:1	0.63	0.70	0.74	0.65	0.70	0.73	0.62	0.41	**0.61**	0.88	0.81	0.62	0.00	0.04
C12:1	0.39	0.48	0.57	0.57	0.71	0.68	0.47	0.16	0.88	**0.63**	0.82	0.63	-0.03	0.00
C14:1	0.45	0.49	0.53	0.47	0.60	0.60	0.50	0.26	0.90	0.93	**0.67**	0.83	0.22	0.23
C16:1	0.61	0.61	0.57	0.48	0.54	0.52	0.71	0.31	0.54	0.53	0.46	**0.49**	0.43	0.37
C18:1	0.82	0.86	0.84	0.72	0.71	0.76	0.79	0.60	0.79	0.66	0.66	0.72	**0.52**	0.48
CLA	0.39	0.49	0.58	0.61	0.69	0.66	0.42	0.09	0.57	0.62	0.50	0.66	0.66	**0.56**

^1^Heritabilities are shown in bold on the diagonal, genetic correlations below the diagonal and residual correlations above diagonal; ^2^In g/kg milk; ^3^Time-series standard errors for the variance components and correlations ranged from 0.0007 to 0.0091 and posterior standard deviations for the variance components and correlations ranged between 0.018 and 0.211.

### Inductive causation (IC)

**Figure 1:**
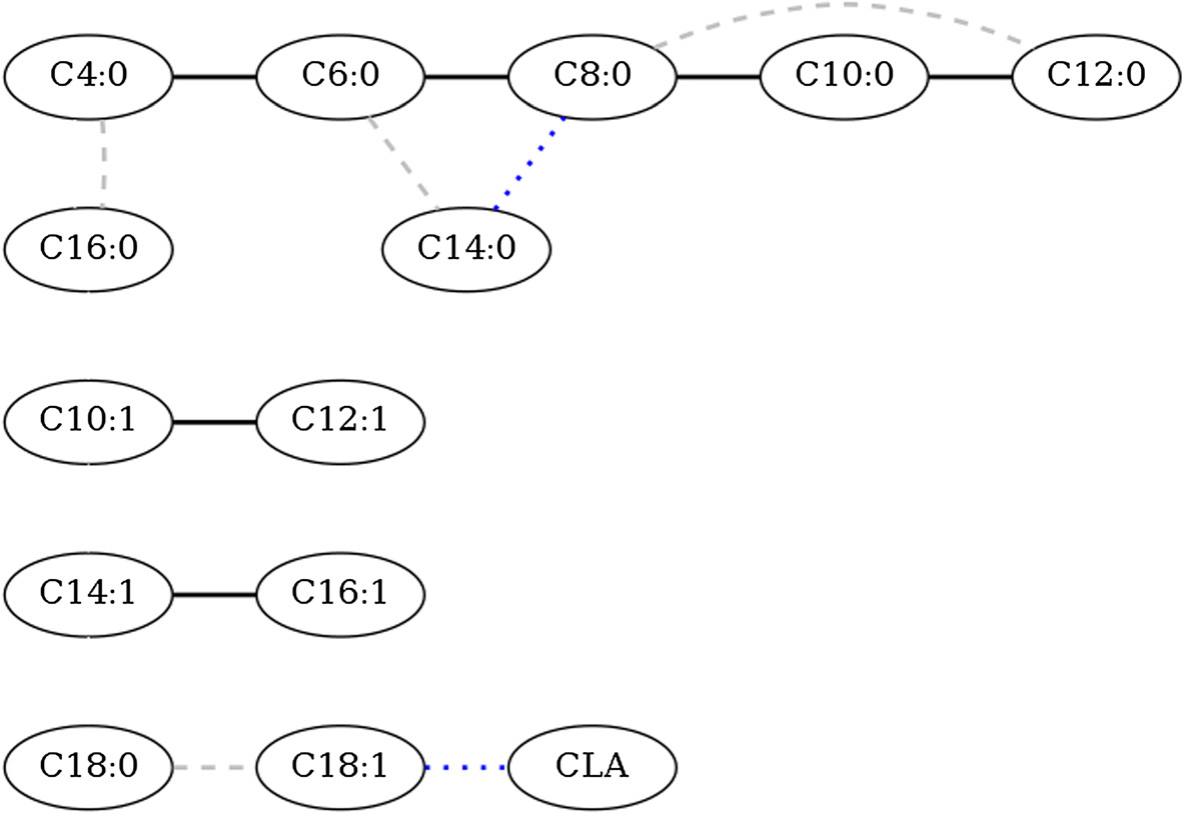
**Network obtained from the inductive causation (IC) algorithm with different highest posterior density (HPD) intervals.** The connections obtained with a HPD interval of 95% and 90% are given in black solid lines, with a HPD interval of 85% in grey dashed lines, and with a HPD interval of 80% in blue dotted lines.

The IC algorithm based on the 95% HPD interval retrieved the undirected network presented in Figure 1 (black solid edges). Consecutive fatty acids C4:0, C6:0, C8:0, C10:0 and C12:0 formed a path of connected nodes. The fatty acids C10:1 and C12:1, as well as C14:1 and C16:1, were also connected to each other. The HPD interval content was reduced to see if there were additional less strong connections between the fatty acids, which may give better results if posterior distributions are not very sharp [13,25]. Reducing the HPD to a probability of 90% resulted in the same network as the HPD interval of 95% (black solid edges in Figure 1). Reducing the HPD interval to 85% resulted in four additional edges: between C4:0 and C16:0, between C6:0 and C14:0, between C8:0 and C12:0, and between C18:0 and C18:1 (grey dashed edges in Figure 1). Reducing the interval further to 80% resulted in two additional edges: between C8:0 and C14:0 and between C18:1 and CLA (blue dotted edges in Figure 1).

No unshielded colliders were recovered from the data in step 2 of the IC algorithm. Therefore, step 3 of the IC algorithm did not result in any additional edge orienting and the resulting network remained undirected.

### Structural equation model (SEM)

A SEM was used to quantify the causal relationships between the milk fatty acids based on a causal structure that was chosen based on the outputs of the IC algorithm. Since a fully oriented structure is required to specify a SEM, the undirected network obtained with the 95% HPD interval (Figure 1, black solid edges) was oriented according to prior biological knowledge about the sequence in which the fatty acids are synthesized in the mammary gland. In this sense, the path C4:0—C6:0—C8:0—C10:0—C12:0 agreed with the *de novo* synthesis of milk fatty acids. According to the *de novo* synthesis, C4:0 should precede C6:0, C6:0 should precede C8:0, and so on. On this basis, the path C4:0—C6:0—C8:0—C10:0—C12:0 could be directed from C4:0 to C12:0, that is C4:0 *→* C6:0 *→* C8:0 *→* C10:0 *→* C12:0. The five traits involved in this path were analysed with both a multi-trait model and a SEM. Both models were compared in terms of fit and parameter inferences.

**Figure 2:**
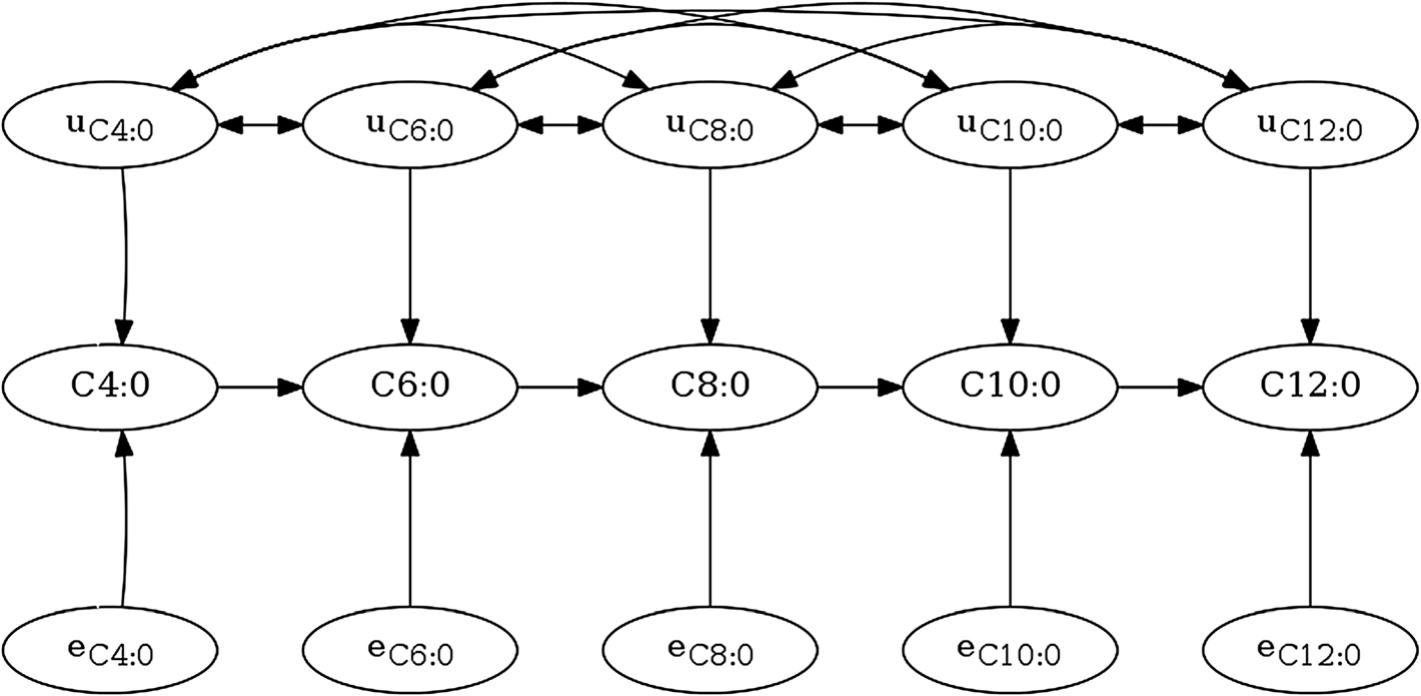
**The fitted causal structure of the structural equation model.** The edges in the fitted structure represent the causal relations for the observed variables (C4:0-C12:0), with independent residuals (e_C4:0_-e_C12:0_) and correlated additive genetic effects (u_C4:0_-u_C12:0_).

The causal network chosen for the SEM shown in Figure 2 resulted in the following structure for the **Λ**-matrix:

$$\Lambda =\left[\begin{array}{ccccc} 0 & 0 & 0 & 0 & 0\\ \lambda_{C6:0,C4:0} & 0 & 0 & 0 & 0\\ 0 & \lambda_{C8:0,C6:0} & 0 & 0 & 0\\ 0 & 0 & \lambda_{C10:0,C8:0} & 0 & 0\\ 0 & 0 & 0 & \lambda_{C12:0,C10:0} & 0 \end{array}\right].$$

**Figure 3:**
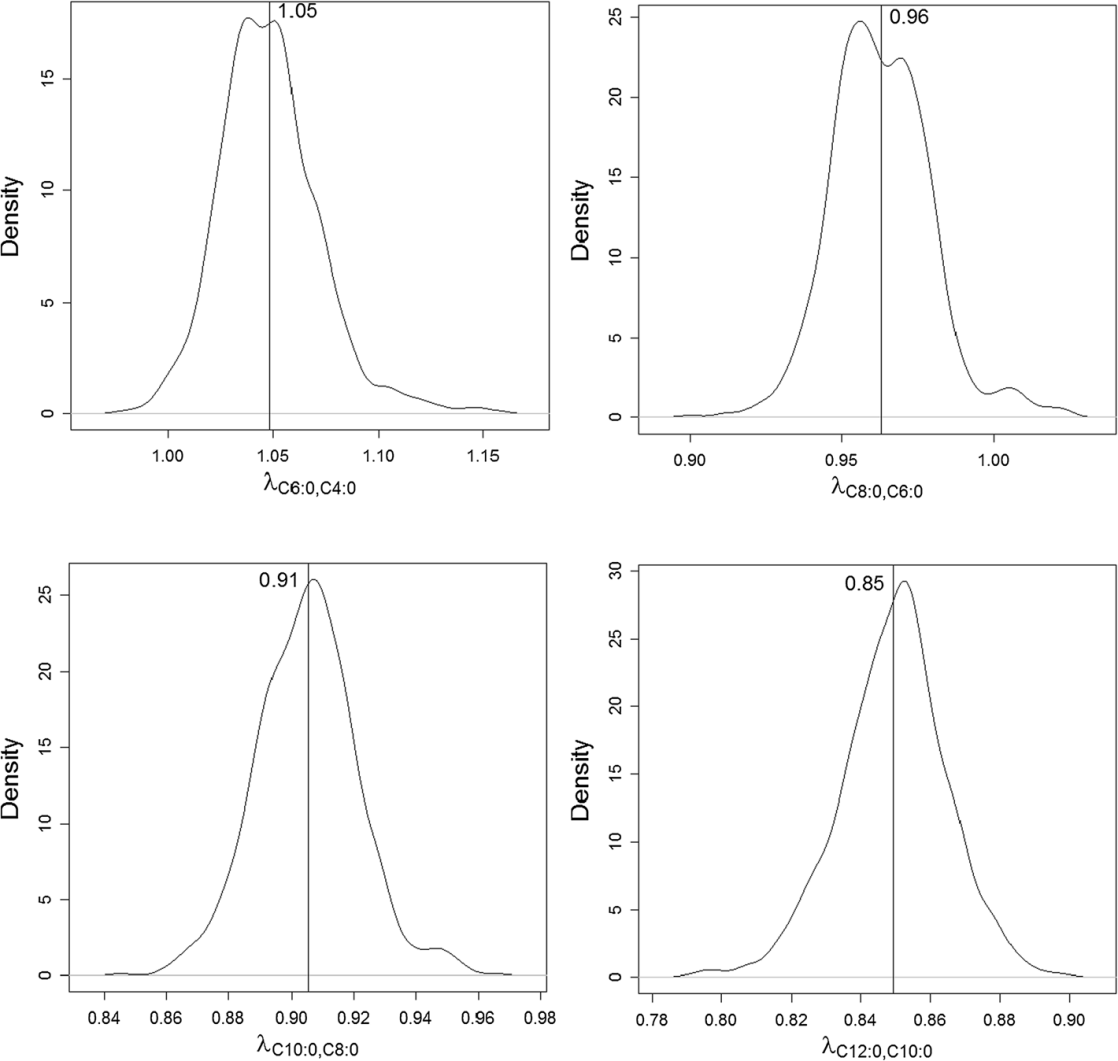
Posterior densities of structural coefficients for the fitted causal structure of the structural equation model.

The posterior densities of the structural coefficients that resulted from the SEM are in Figure 3. The posterior means of these parameters ranged from 0.85 to 1.05 (Figure 3).

Table 3 shows the posterior means for the parameters from both the multi-trait model and the SEM for C4:0, C6:0, C8:0, C10:0 and C12:0. As pointed out by Valente et al. [26], genetic effects from multi-trait models and SEM have different meanings: the latter represent direct genetic effects (i.e., genetic effects that are not mediated by other traits in the causal network), while the former represent overall genetic effects (i.e., a combination of all direct and indirect genetic effects on each trait). Model specific genetic (co)variances refer to the (co)dispersion of the genetic effects of each model, and therefore have distinct meanings as well. The posterior means of the genetic variances of the multi-trait model for C4:0, C6:0, C8:0, C10:0 and C12:0 were fairly similar to each other (i.e., between 0.360 for C4:0 and 0.276 for C12:0), while the posterior means of the SEM genetic variances for C4:0, C6:0, C8:0, C10:0 and C12:0 showed a gradual decrease (i.e., 0.460 for C4:0, 0.114 for C6:0, 0.073 for C8:0, 0.066 for C10:0 and 0.004 for C12:0), indicating that indirect genetic effects from upstream traits were gradually explaining a larger portion of genetic variability. Such reduction was even stronger for the SEM residual variance. Statistically, this result was expected because conditioning on the strongly correlated traits in the SEM removed a large proportion of the observed variance. On the basis of the given causal structure, this indicates that the variability of each of these fatty acids can be almost fully explained by the conditioning (parent) fatty acid. The posterior means of the genetic and residual variances of C4:0 for the SEM were similar to those for the multi-trait model, because C4:0 was not conditioned on any of the other traits. The posterior means of the genetic correlations from the SEM refer to the genetic covariance that is left after conditioning on the appropriate fatty acids, i.e., it expresses the correlation between direct genetic effects for each trait. For that reason, the SEM genetic correlations were different from the correlations estimated with the multi-trait model.

**Table 3:** Posterior means of the variance components for the multi-trait and the structural equation model of C4:0 to C12:0

**Variance component**^**1**^	**Multi-trait**			**SEM**		
	**Mean**	**SD**^**2**^	**Time-series SE**^**3**^	**Mean**	**SD**^**2**^	**Time-series SE**^**3**^
$\sigma_{e}^{2}$ C4:0	0.549	0.108	0.003	0.455	0.091	0.002
$\sigma_{e}^{2}$ C6:0	0.606	0.102	0.004	0.003	0.002	0.000
$\sigma_{e}^{2}$ C8:0	0.599	0.100	0.004	0.000	0.000	0.000
$\sigma_{e}^{2}$ C10:0	0.560	0.102	0.004	0.006	0.002	0.000
$\sigma_{e}^{2}$ C12:0	0.459	0.087	0.003	0.059	0.004	0.000
*r*_*e*_ C4:0,C6:0	0.938	0.019	0.001	.	.	.
*r*_*e*_ C4:0,C8:0	0.885	0.046	0.001	.	.	.
*r*_*e*_ C4:0,C10:0	0.808	0.084	0.002	.	.	.
*r*_*e*_ C4:0,C12:0	0.754	0.101	0.003	.	.	.
*r*_*e*_ C6:0,C8:0	0.950	0.014	0.000	.	.	.
*r*_*e*_ C6:0,C10:0	0.906	0.036	0.001	.	.	.
*r*_*e*_ C6:0,C12:0	0.859	0.053	0.002	.	.	.
*r*_*e*_ C8:0,C10:0	0.950	0.014	0.000	.	.	.
*r*_*e*_ C8:0,C12:0	0.911	0.028	0.001	.	.	.
*r*_*e*_ C10:0,C12:0	0.934	0.017	0.001	.	.	.
$\sigma_{g}^{2}$ C4:0	0.360	0.151	0.005	0.460	0.122	0.002
$\sigma_{g}^{2}$ C6:0	0.325	0.143	0.005	0.114	0.023	0.001
$\sigma_{g}^{2}$ C8:0	0.310	0.140	0.005	0.073	0.009	0.000
$\sigma_{g}^{2}$ C10:0	0.319	0.141	0.005	0.066	0.008	0.000
$\sigma_{g}^{2}$ C12:0	0.276	0.121	0.004	0.026	0.005	0.000
*r*_*g*_ C4:0,C6:0	0.855	0.074	0.002	-0.440	0.123	0.004
*r*_*g*_ C4:0,C8:0	0.675	0.157	0.005	-0.417	0.116	0.004
*r*_*g*_ C4:0,C10:0	0.424	0.237	0.007	-0.400	0.109	0.003
*r*_*g*_ C4:0,C12:0	0.331	0.255	0.008	-0.084	0.089	0.002
*r*_*g*_ C6:0,C8:0	0.863	0.069	0.002	0.761	0.033	0.001
*r*_*g*_ C6:0,C10:0	0.697	0.148	0.004	0.730	0.036	0.001
*r*_*g*_ C6:0,C12:0	0.617	0.179	0.006	0.160	0.154	0.004
*r*_*g*_ C8:0,C10:0	0.862	0.071	0.002	0.692	0.036	0.001
*r*_*g*_ C8:0,C12:0	0.805	0.102	0.003	0.152	0.147	0.004
*r*_*g*_ C10:0,C12:0	0.899	0.052	0.002	0.148	0.142	0.004

^1^$\sigma_{e}^{2}$ is residual variance, $\;\sigma_{g}^{2}$ is genetic variance, *r*_*e*_ is residual correlation, *r*_*g*_ is genetic correlation; ^2^SD is the posterior standard deviations of the component; ^3^Time-series SE is the time-series standard error of the component.

The DIC for the multi-trait model for C4:0, C6:0, C8:0, C10:0 and C12:0 was -21 083, while the DIC for the SEM using the structure depicted in Figure 2 was -32 406, indicating that the studied structure is plausible [24]. This lower DIC for the SEM is partly due to a lower penalty for model complexity in the DIC for the SEM. Although the SEM introduces sources of covariance from the causal associations, the residuals of the SEM were assumed to be uncorrelated, which resulted in a model that was more parsimonious than the multi-trait model. This lower penalty for model complexity was reflected by a lower effective number of parameters (5152 for the SEM and 6829 for the multi-trait model).

## Discussion

The aim of this study was to explore causal networks of milk fatty acids by applying the IC algorithm in a mixed model context. Undirected acyclic graphs were obtained for several HPD intervals. A subset of five fatty acids formed a structure that could be directed based on prior knowledge and this structure was then used in a SEM to quantify the relationships between them.

### Direction of network based on prior knowledge

The networks obtained for the 14 fatty acids were undirected. Based on the known sequence of the synthesis of fatty acids, edges could be directed without creating cycles or unshielded colliders that were not supported by the data. Fatty acid C4:0 precedes C6:0, which in turn precedes C8:0 and so on in the *de novo* synthesis, which led us to suggest that the path containing C4:0, C6:0, C8:0, C10:0 and C12:0 is directed from C4:0 to C12:0. This means that the final network is not completely data-driven. However, the structure for this subset of fatty acids that is plausible based on biological knowledge does not have colliders, so the fact that the algorithm could not detect directions was expected. Therefore, not finding any unshielded colliders among these fatty acids supports the hypothesis of a path directed from C4:0 to C12:0.

### Linearity

The search space does not contain cyclic structures and non-linear relations are also not considered in the specific application presented here. Instead, as in most studies, it was assumed that relationships between traits were linear but in reality they could be non-linear. In contrast to the assumptions of the adapted IC algorithm applied here, SEM can be extended to include, for instance, interactions, feedback mechanisms (cyclic relations), quadratic terms or polynomials to determine which model fits the data best [27,28]. In addition, the search algorithm could make decisions based on alternative tests for conditional independence instead of on partial correlations [3].

### Causal sufficiency assumption

Connections between variables are often referred to as causal relations, but the only widely accepted method for declaring causation between two variables is a randomised experiment. This involves random assignment of each subject to different treatment groups, coupled with random assignment of treatment level to each group, and results in averaging out potential sources of confounding effects. In the analysed data, variables that act as confounders are not averaged out, but when they are measured, they can be included in the model to correct for this confounding effect. Based on model assumptions, causation can be inferred, but because of the impossibility of declaring with absolute certainty that there are no additional unmeasured causal variables, these assumptions cannot be guaranteed. The IC algorithm is based on the assumption that there are no hidden variables that affect more that one of the variables considered in the model, which is called the causal sufficiency assumption [29]. If this assumption does not hold, there may be direct connections between variables that are not causal relations but that are due to other sources, such as common hidden causes. Although a SEM does not require this assumption, it is commonly applied for the sake of model identifiability.

### Comparison between the network obtained and known biological networks

Metabolic pathways involved in the synthesis of milk fatty acids, such as *de novo* synthesis, desaturation and biohydrogenation, could be reflected in the structure provided by the IC algorithm. In the following, the network obtained with the IC algorithm will be compared with known metabolic pathways of milk fat synthesis. For this comparison, two aspects should be noted. First, the variables studied here are fatty acids excreted in the milk, which are not necessarily the same variables as the corresponding fatty acids involved in the milk fat synthesis pathways, e.g., C6:0 measured in milk is not the same as a C6:0 in the elongation cycle of the *de novo* synthesis being transformed into C8:0. This is especially important considering that the SEM expresses the causal effect between fatty acids excreted in the milk, which are the recorded phenotypes. These causal effects reflect expected results of (ideal) external interventions. However, the expected consequences of modifying a fatty acid that is excreted in the milk on other fatty acids may not be the same as the consequences of manipulating the amount of a specific fatty acid during synthesis in the mammary gland.

The second aspect is that the proposition that an object B originates from an object A does not necessarily imply that causal effects between measurements *a* and *b* made respectively on A and B must be directed as *a → b*. Therefore, if fatty acid B originates from fatty acid A in the synthesis process in the mammary gland, measurements of the concentration of these fatty acids in the milk (*a* and *b*) are not necessarily directed as *a → b* if they are causally connected. So it is possible that edges may actually have alternative directions, and that is not a strict contradiction of known biochemical paths. For example, inoculating C8:0 in the mammary gland could affect the amount of C6:0 released in the milk, which would be an effect that is opposite to the description of how C8:0 originates from C6:0, but does not deny that C8:0 originates from C6:0. Although one could defend such an alternative structure (and other statistically equivalent ones), the structure chosen to fit the model is credible given its expected intervention outcome. For instance, the chosen structure expresses that if C8:0 is inoculated in the mammary gland, then C4:0 and C6:0 would remain the same, but such intervention would affect C10:0 and also, indirectly, C12:0. This is compatible with a scenario in which C8:0 is inoculated: C4:0, and C6:0 would be normally produced since their synthesis occurs earlier in the cycle, and less C8:0 would be released in the milk, since its concentration is already high due to the inoculation (in case there is some regulation of fatty acids production by the concentration of free fatty acids). This would leave more “substrate” remaining within the cycle for the subsequent fatty acids and would result in increasing C10:0, and so forth. This is compatible with the causal meaning of the chosen structure (and the inferred structural coefficients, if they are positive). It should be noted that in this case, the meaning of the graph C4:0 → C6:0 → C8:0 → C10:0 → C12:0 depends on whether it is interpreted as a biochemical pathway that shows how fatty acids are originated or as a SEM that involves the concentrations of such fatty acids, although both interpretations could be represented with the same nodes and directed connections. For the structure of the SEM fitted (C4:0 *→* C6:0 *→* C8:0 *→* C10:0 *→* C12:0), directions were chosen that mirror the *de novo* pathway, because it is plausible (although not necessary) based on how the fatty acids are generated and on that basis, if the underlying causal structure indeed reflected the metabolic pathway, the expected output of the search algorithm would be exactly C4:0—C6:0—C8:0—C10:0—C12:0.

#### De novo synthesis

Short and medium chain saturated fatty acids (C4:0-C14:0 and about half of the C16:0 present in milk) are produced in the *de novo* synthesis pathway. In this metabolic pathway, the carbon chain is elongated in a sequential cyclic reaction from acetate and β-hydroxybutyrate until a C16:0 fatty acid is formed by fatty acid synthase in the mammary gland e.g., [30,31]. In the bovine, all intermediate fatty acids can leave the elongation cycle by a chain termination mechanism [32] and thus end up in bovine milk. The path from C4:0 to C12:0 that was obtained from the IC algorithm with a HPD interval of 95% (black solid edges in Figure 1) mirrored this *de novo* synthesis. One could argue that the path obtained from the IC algorithm should also include C14:0 and C16:0 but part of C14:0 and C16:0 originate from the cows’ diet, which might have reduced the degree of association with the remaining pathway, thus leading the search algorithm to declare them disconnected from the remaining variables, i.e. excluding them from the pathway. The structural coefficients that were estimated using the SEM with the causal structure C4:0 *→* C6:0 *→* C8:0 *→* C10:0 *→* C12:0 indicate that if C4:0 increases 1 g/kg milk, then C6:0 would respond by increasing 1.05 g/kg milk (Figure 3). However, the molar mass of C6:0 is 1.32 times the molar mass of C4:0, so although the relationship is nearly one to one unit-wise, is less than one based on molar mass. The structural coefficients *λ*_*C*10 : 0,*C*8 : 0_ and *λ*_*C*12 : 0,*C*10 : 0_ were slightly lower than *λ*_*C*6 : 0,*C*4 : 0_ and *λ*_*C*8 : 0,*C*6 : 0_, possibly because a small part of C10:0 and C12:0 is desaturated into C10:1 and C12:1 in the mammary gland. These structural coefficients suggest that an intervention that increases the amount of C4:0 secreted in milk would result in an increase in C6:0 secreted in milk and that would in turn result in an increase in C8:0, C10:0 and C12:0 secreted in milk.

#### Desaturation

Medium chain saturated fatty acids (C10:0-C16:0) are desaturated by coenzyme A desaturase 1 (*SCD1*) into their equivalent mono-unsaturated fatty acids (C10:1-C16:1) in the mammary gland [31,33]. Structures that mirror this desaturation pathway (e.g., C10:0 *→* C10:1) were not recovered by the IC algorithm (Figure 1). The obtained structures (C10:1—C12:1 and C14:1—C16:1) showed that the amount of mono-unsaturated medium chain fatty acids measured in milk are not causally associated with the amount of their equivalent saturated fatty acid, but suggest that the mono-unsaturated medium chain fatty acids may have a common hidden causal variable among them.

#### Biohydrogenation

Long chain fatty acids (half of the C16:0 present in milk and all fatty acids with 18 or more carbons) originate from the diet fed to cows and are biohydrogenated by micro-flora in the rumen into C18:0 and multiple intermediate products [31]. Some edges were recovered between the long chain fatty acids, e.g. between C18:0 and C18:1, and between C18:1 and CLA, which likely represent this biohydrogenation process. These edges were recovered when the HPD interval was relaxed to 80-85%, which indicates weak evidence for these edges (Figure 1).

Reducing the HPD interval resulted in additional edges. The edges that involve long chain fatty acids might be plausible associations but the edges between C4:0 and C16:0, C6:0 and C14:0, C8:0 and C12:0, C8:0 and C14:0 appear to be false positive associations due to the lowered threshold.

To conclude, although the fatty acids were measured when secreted in milk and not during their synthesis in the mammary gland, concentrations of fatty acids in milk mirror some of the metabolic pathways, and resemblance with the *de novo* synthesis pathway obtained most evidence.

### Convergence issues of the multi-trait model

The search for causal structures among a set of variables makes sense if associations exist between them. However, if many traits have strong correlations with each other, fitting multi-trait mixed models may encounter convergence issues, which was the case in the current study. Most milk fatty acids were strongly correlated with each other, both genetically and residually. Fitting a standard multi-trait model for 14 milk fatty acids resulted in slow MCMC convergence, strong auto lag correlations in the chain and thus in a small number of effective samples.

Running the MCMC Bayz 2.1 [22] program using latent variables to reduce the dimensionality of the data improved convergence of the Bayesian multi-trait mixed model. A principal component analysis showed that using four latent variables was reasonable for the multi-trait model with 14 fatty acids. Using latent variables has some effect on the modelled (co)variance structures; because the latent variable model uses less parameters than the full (co)variance matrix, the (co)variance structure is somewhat restricted, similar to using only the main principal components in a principal component analysis or frequentist factor analytic model, e.g., [34]. In this case, the latent variable model used 70 parameters [(4 *latent variables* + 1) × 14 *traits*] for each of the environmental and genetic (co)variance structures, whereas the full (co)variance matrix has 105 parameters. For the multi-trait model for C4:0, C6:0, C8:0, C10:0 and C12:0, two latent variables were used, resulting in 15 parameters and thus no restrictions on the (co)variance matrix. The multi-trait model for C4:0 to C12:0 resulted in the same pathway as the model with 14 traits, suggesting that the restriction in parameters due to latent variables did not influence this particular pathway.

A final measure to improve convergence was to set minimum bounds on the remaining independent variances $\tau_{e_{i}}^{2}$ and $\tau_{u_{i}}^{2}$ for residuals and genetic effects through the prior distributions. These minimum bounds were set at 0.02 (on standardised phenotypes), which implies that heritabilities were constrained to be between 2 and 98%, and that all correlations were forced to remain slightly below 1. These adaptations were required for the model to converge such that this dataset could be explored for causal networks.

### Computation time of the adapted Inductive Causation (IC) algorithm

The approach suggested by Valente et al. [12] is more complex and computationally demanding than the standard use of the IC algorithm and other similar methods that simply work with unconditional point estimates of covariance matrices, not requiring prior model fitting. Although this is appealing in the context of mixed effects SEM, there is a compelling reason to follow the approach of Valente et al. [12] because mixed effects SEM allow direct genetic covariances, which are extra genetic sources of associations among traits, aside from causal effects. Assuming these genetic associations to be absent would be more difficult to accept, since genetics most likely affects multiple traits of a set in a way that is not mediated by other traits in the set. Using the IC algorithm on raw data assumes that these correlated direct genetic effects do not exist and, therefore, requires assumptions that are more difficult to accept. Furthermore, using the output from such an IC analysis in a mixed effects SEM with unstructured genetic covariances implies inconsistency of assumptions in the different analysis steps.

The computation time of the IC algorithm increases rapidly with an increasing number of analysed traits, because of the increasing number of partial correlations to be tested. The IC algorithm required testing the partial correlations between each pair of fatty acids conditional on all possible subsets of the remaining fatty acids. With 14 traits there are 91 distinct pairs of traits [*n* × (*n* - 1)/2] and 4096 possible conditioning sets (2^n-2^), leading to 372 736 partial correlations to be calculated for each posterior sample of the residual (co)variance matrix (i.e., 2^*n* - 1^ × *n* × (*n* - 1)/2). In addition, the size of the posterior sample also affects computation time. Additional thinning of the MCMC speeds up computation time for the adapted IC algorithm. Parallel computing would be a promising strategy to reduce the computation time of the algorithm. However, other refinements to the method used here will be needed when the number of variables increases strongly, for instance with high-throughput gene expression data, such as microarray or RNA-seq.

### Possibilities

Correlations between traits play a role in livestock management practices. These correlations can result from different causal relationships, such as direct or indirect causal effects between traits, or from a common causal parent, or even from a combination of these. The concentrations of fatty acids in milk are clearly correlated, but the partial correlations indicate that only a few are directly connected in the network. Even an undirected structure is informative and reveals direct and indirect associations between variables. Nonetheless, prior knowledge may be used to orient additional edges, and resulting causal inferences can then be confirmed with additional data and studies. Representing the associations between traits with networks may provide better insights into the underlying biological mechanisms and offer opportunities for management tools to focus on pathways instead of correlations. Response to interventions applied to a biological system can be predicted using SEM. Shifting the focus from correlation matrices to causal diagrams might result in faster and better understanding of responses to interventions. The principles of the IC algorithm and SEM can also be used to investigate gene regulatory networks in gene expression studies [35-37]. Understanding the relationships between genes can, for instance, identify targets for intervention that could contribute to the development of therapies for certain diseases.

## Conclusions

Application of the adapted IC algorithm proposed by Valente et al. [12] resulted in an undirected network for the 14 milk fatty acids studied. The pathway from C4:0 to C12:0 reflected the *de novo* synthesis pathway of short and medium chain saturated fatty acids. By using prior biological knowledge, directions were assigned to that part of the network and the resulting structure was used to fit an SEM. The edges between C10:1 and C12:1 and between C14:1 and C16:1 did not correspond to associations reported in the literature, which might be due to a common hidden causal variable. Other expected relations based on biological knowledge were not found or were detected only when the HPD interval was relaxed.

The output of the IC algorithm suggested causal relations between the studied traits. This changes the focus from marginal associations between traits to direct relationships that may result in changes when external interventions are applied. The causal structure can give more insight into underlying mechanisms and the SEM can predict conditional changes due to such interventions.

## Competing interests

The authors declare that they have no competing interests.

## Authors’ contributions

ACB edited the data, carried out the analysis, prepared and drafted the manuscript. BDV participated in the design of the study, was involved in discussions, and helped to draft the manuscript. LLGJ developed and adapted the software used for the Bayesian multi-trait analysis, was involved in discussions, and helped to draft the manuscript. HB participated in the coordination, was involved in discussions and helped to draft the manuscript. GJMR participated in the design of the study and the coordination, was involved in discussions, and helped to draft the manuscript. All authors read and approved the final manuscript.
